# Nasal myiasis under direct bronchoscopic visualization

**DOI:** 10.1002/rcr2.892

**Published:** 2021-12-22

**Authors:** Ahel El Haj Chehade, Jordan Metcalf, Blake Jacobs

**Affiliations:** ^1^ Department of Pulmonary, Critical Care and Sleep Medicine The University of Oklahoma Health and Sciences Center Oklahoma City Oklahoma USA

**Keywords:** bronchoscopy, nasal myiasis, opportunistic infections, septic shock

## Abstract

This is a video showing a case of nasal myiasis under direct visualization with flexible bronchoscopy in a patient admitted with septic shock and metastatic prostate cancer. Microbiology revealed *Lucilia sericata* larvae.

## Graphical abstract

This video shows a case of nasal myiasis under fibreoptic bronchoscopic visualization in a patient admitted with septic shock and metastatic prostate cancer.

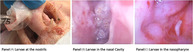

## VIDEO

**FIGURE 1:**
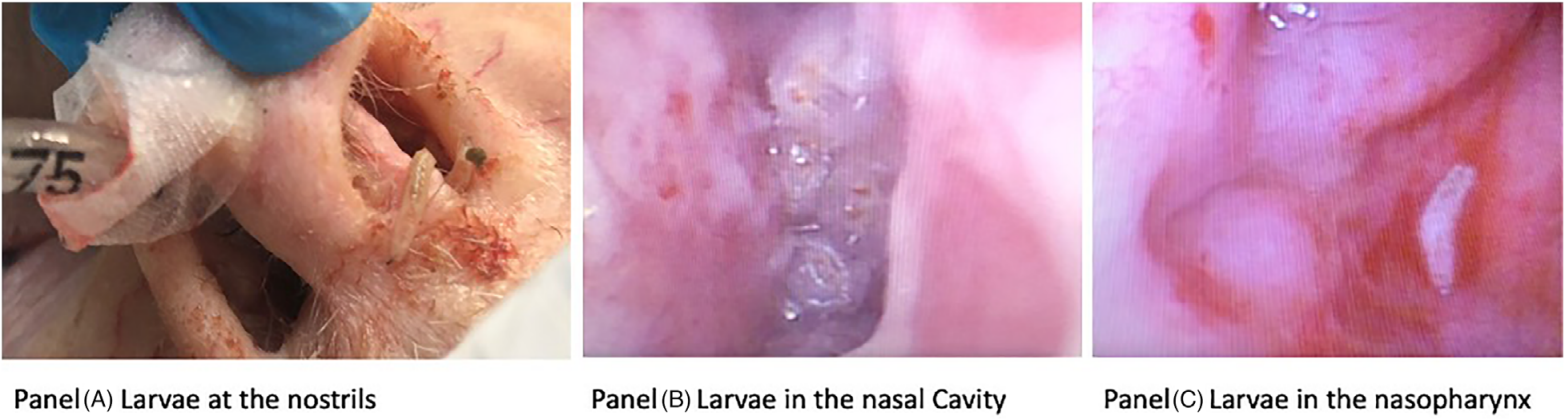
(A) Larvae at the nostrils. (B) Larvae in the nasal cavity. (C) Larvae in the nasopharynx

Nasal myiasis is rare in developed countries. The patient in this report is a 72‐year‐old man who was a long‐term nursing home resident with a history of metastatic prostate cancer, congestive heart failure and chronic kidney disease. He was admitted to the hospital for altered mental status and fever, and was found to have urosepsis. Two days after admission, his condition deteriorated, and he required intubation, mechanical ventilation and vasopressor therapy for worsening septic shock. Directly, after intubation, maggots were noted to come out of both nostrils (Figure 1A) and endotracheal tube (Video 1, Part 1 ). A nasal inspection with flexible bronchoscopy revealed multiple larvae consistent with nasal myiasis (Figure 1B,C, Video 1, Part 2 ). Bronchoscopy and a computed tomography (CT) chest ruled out lung involvement. CT scan of the brain and sinuses did not reveal any central nervous system or sinus involvement. Eye examination and skin examination were unremarkable. Nasal larvae retrieval and suctioning was attempted with success. Microbiological analysis of the larvae revealed *Lucilia sericata* myiasis. This species in known to be endemic in Oklahoma. The disease course is usually benign and rarely invasive. We believe that our patient being immunocompromised, and bed ridden might have been predisposing risk factors. From the life cycle of the species and our patient having no nasal symptoms on admission, we concluded that the infection was acquired in the nursing home. The nursing home, the hospitals infection control department and the state's department of health were alerted to take appropriate measures. Usually, the treatment involves larvae retrieval and occasionally topical or oral helminthic agent. Our patient was started on oral ivermectin. Unfortunately, the patient passed away from his critical illness and multi‐organ failure.

**VIDEO 1:** Part 1: Larva in the endotracheal tube. Part 2: Nasal bronchoscopy showing multiple larvae

## CONFLICT OF INTEREST

None declared.

## AUTHOR CONTRIBUTION

Ahel El Haj Chehade participated in collecting and editing images and videos, and participated in drafting and revising the manuscript. Blake Jacobs participated in collecting and editing images and videos, and participated in revising the manuscript. Jordan Metcalf supervised the project and participated in revising and editing the manuscript.

## ETHICS STATEMENT

The authors declare that appropriate written informed consent was obtained for the publication of this manuscript and accompanying images.

## Data Availability

Data sharing is not applicable to this article as no new data were created or analyzed in this study.

